# Identification of Tyrosyl Oleate as a Novel Olive Oil Lipophenol with Proliferative and Antioxidant Properties in Human Keratinocytes

**DOI:** 10.3390/antiox10071051

**Published:** 2021-06-29

**Authors:** Cinzia Benincasa, Chiara La Torre, Alessia Fazio, Enzo Perri, Maria Cristina Caroleo, Pierluigi Plastina, Erika Cione

**Affiliations:** 1 CREA Research Centre for Olive, Fruit and Citrus Crops, 87036 Arcavacata di Rende, CS, Italy; cinzia.benincasa@crea.gov.it (C.B.); enzo.perri@crea.gov.it (E.P.); 2 Department of Pharmacy, Health and Nutritional Sciences, University of Calabria, 87036 Arcavacata di Rende, CS, Italy; latorre.chiara@libero.it (C.L.T.); alessia.fazio@unical.it (A.F.); mariacristina.caroleo@unical.it (M.C.C.); erika.cione@unical.it (E.C.)

**Keywords:** polyphenol, lipophenol, phenolipid, tyrosol, hydroxytyrosol, fatty acid ester, antioxidant, proliferation, reactive oxygen species, reactive oxygen species (ROS)

## Abstract

Lipophenols are an emerging subclass of phenolic compounds characterized by the presence of a lipid moiety. Recently, hydroxytyrosyl oleate (HtyOle), a derivative of hydroxytyrosol, has been identified in olive oil and by-products. Furthermore, HtyOle possesses anti-inflammatory, antioxidant, and tissue regenerating properties. In this work, the potential occurrence of tyrosyl oleate (TyOle) in olive oil was investigated based on the hypothesis that its precursors tyrosol and oleic acid, both present in relatively high amount can be coupled together. Moreover, TyOle effects have been investigated in human keratinocytes to verify its proliferative and antioxidant properties. The quantitative determination of TyOle was carried out by the external standard method in liquid chromatography coupled with mass spectrometry (LC/MS), in negative mode using multiple reaction monitoring (MRM). The proliferative properties of TyOle on immortalized human keratinocytes (HaCat) were evaluated by 3-(4,5-dimethylthiasol-2-yl)-2,5-diphenyltetrazolium bromide (MTT) assay. Morphological changes were observed by fluorescent staining with phalloidin (for F-actin) or 4,6-diamidino-2-phenylindole (DAPI, for chromatin) dye. The antioxidant activity was assessed at the level of production of mitochondrial reactive oxygen species (ROS) induced with UV exposure. TyOle was identified in all the oil samples investigated. Interestingly, TyOle concentration was higher in defective or low-quality oils than in extra virgin oils. The formation of TyOle likely occurs during the crushing and kneading processes and its concentration is related to the increase of rancidity and of the concentration of free precursors. Herein we show that TyOle induced an increase in the viability of HaCat cells and cytoskeletal remodeling.

## 1. Introduction

**Figure 1:**
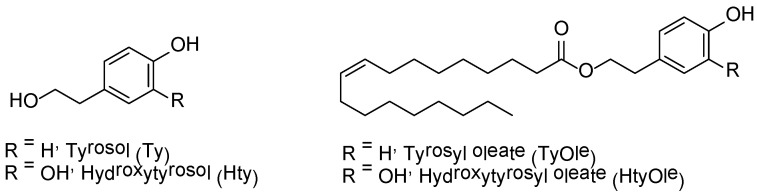
Structure of tyrosol (Ty), hydroxytyrosol (Hty), tyrosyl oleate (TyOle) and hydroxytyrosyl oleate (HtyOle).

Olive oil represents the main source of fat in the Mediterranean diet and represents a valuable food product from both nutritional and economic point of view. Its worldwide consumption has increased in the last five years [1]. The nutritional value and the health promoting effects of olive oil rely on its favorable nutrient composition, including oleic acid (OA) as the most abundant fatty acid and fat-soluble vitamins and the presence of phenolic compounds [2,3,4,5]. The latter are recognized in contributing to the positive health effects related to the consumption of extra virgin olive oil (EVOO) [6,7]. Noteworthy, the Regulation 432/2012 of the EU approved the claim “olive oil polyphenols contribute to the protection of blood lipids from oxidative stress” based on the scientific opinion of the European Food Safety Authority (EFSA) that “a daily intake of 20 g of olive oil, which contains at least 5 mg of hydroxytyrosol and its derivatives (e.g., oleuropein and tyrosol) provides the expected beneficial effects” [8,9]. Tyrosol (2-(4-hydroxyphenyl)ethanol, Ty, Figure 1) and hydroxytyrosol (2-(3,4-dihydroxyphenyl)ethanol, Hty, Figure 1) are among the main phenolic compounds found in olive and olive oil. They occur in their free form as well as in the esterified forms, mainly as secoiridoid derivatives (ligstroside and oleuropein, respectively) [10,11] or acylated, as in the case of hydroxytyrosyl acetate [12].

Recently, hydroxytyrosyl oleate (HtyOle, Figure 1), a derivative of Hty characterized by a lipophilic nature due the presence of an oleic acid fatty chain, has been identifified and quantified in EVOO [13,14], as well as in the by-products of olive oil industry, pomace and olive mill waste waters (OMWWs) [15]. Moreover, HtyOle has been found to possess anti-inflammatory, antioxidant, as well as tissue-regenerating properties [14,15]. The conjugation of fatty acids with active cosmetic ingredients leads to the formation of fatty derivatives with enhanced skin penetration ability with respect to small hydrophilic compounds, thus increasing their antimicrobial, antioxidant, or anti-ageing activities [16,17,18]. In this work, we investigated the possibility that another lipophenol (tyrosyl oleate, TyOle, Figure 1) could occur in olive oil as a result of the reaction between tyrosol and oleic acid, both occurring in relatively high amount. Moreover, proliferative and antioxidant properties of TyOle have been investigated in human keratinocyte cell line to explore the possibility that TyOle could have tissue regenerating properties like its congener HtyOle.

## 2. Materials and Methods

### 2.1. Chemicals and Reagents

Tyrosol, oleic acid (OA), and methyl oleate, *t*-butanol, methanol, dimethyl sulfoxide (DMSO) and formic acid (LC/MS grade) were purchased from Sigma-Aldrich (Milan, Italy). Novozym^®^435 (immobilized *Candida antarctica* Lipase B) was from Novozymes (Bagsværd, Denmark). *n*-Hexane and acetone (analytical grade) were supplied from Carlo Erba Reagenti (Milan, Italy). Ultrapure water (resistivity of 18.2 MΩ·cm) was obtained from a Milli-Q plus system (Millipore, Bedford, MA, USA). Dulbecco’s modified Eagle’s medium (DMEM), fetal bovine serum (FBS), l-glutamine, penicillin/streptomycin, and paraformaldehyde (PFA) were supplied from Thermo Fisher Scientific (Waltham, MA, USA).

### 2.2. Instrumentations

NMR analyses were performed at 25 °C on a Bruker AC 300 spectrometer at 300 MHz and 75 MHz for ^1^H and ^13^C NMR, respectively. CDCl_3_ and tetramethylsilane were used as the solvent and the internal standard, respectively. HPLC analyses were performed using an Agilent Technologies 1200 series liquid chromatography system equipped with G1379B degasser, G1312A pump, and G1329A autosampler. Mass spectra were obtained in the negative mode using an API 4000 Q-Trap (MSD Sciex Applied Biosystem, Framingham, MA, USA) mass spectrometer equipped with ion max source with ESI probe (ion spray voltage (IS) −4500 V; curtain gas, 20 psi; entry potential (EP), −11 eV; declustering potential (DP), −125 eV; collision energy (CE), −20 eV and collision exit potential (CXP), −16 eV) through direct infusion (10 μL·min^−1^) of a methanol solution of TyOle (5 μg·mL^−1^).

### 2.3. Chemistry

The synthesis of TyOle was achieved according to our previously reported enzymatic procedure [15]. Tyrosol (1.6 mmol) reacted for 24 h in an orbital shaker at 50 °C with methyl oleate (3.2 mmol), with Novozym^®^435 (200 mg) as the catalyst, in *t*-butanol (2 mL). After cooling down the temperature and filtration of the enzyme, the solvent was removed by reduced pressure evaporation. TyOle was purified by means of column chromatography (SiO_2_, *n*-hexane-acetone as the eluent). Spectroscopic data agreed with those available in the literature [19], and purity (>98%) was assessed by HPLC.

### 2.4. Olive and Olive Oil Samples

*Olea europaea* L. drupes (cultivar: Carolea, Cassanese, Coratina, Grossa di Spagna and Nocellara del Belice) were hand-collected in the experimental field of the CREA Research Centre for Olive, Fruit and Citrus Crops (Italy) during the campaign 2017–2018. Extra virgin olive oils (from Carolea, Dolce di Rossano e Nocellara del Belice cultivars) were produced by the company “La Molazza” located in Cantinella di Corigliano (Italy) during the campaign 2017–2018. Defective olive oils, produced in previous campaigns, were stored in CREA Research Centre for Olive, Fruit and Citrus Crops (Italy). Dried destoned virgin olive pomace (DDVOP) was obtained from the dehydration of olive pomace by the use of a high-temperature oven. The dried destoned virgin olive pomace oil (DDVOPO) was extracted from the latter by the use of solvents.

### 2.5. Extraction of Phenolics from Olives and Olive Oils

#### 2.5.1. Method 1

The method used by Bianco et al. for the isolation and the characterization of TyOle from the drupes (cultivar: Cassanese) was used [20]. Olive pulp (100 g) or olive oils (2 g) were mixed with a methanol–acetone (1/1; *v*/*v*) solution containing 0.5% sodium metabisulfite. The mixtures were sonicated and centrifuged for five times. Supernatants were collected, concentrated in vacuo and the residue was dissolved in acetone. After further centrifugation, the supernatants were concentrated in vacuo and the residue was dissolved in acidic water (pH 2). A final centrifugation was carried out in order to separate the phenolic-rich phase from the aqueous phase. The crude sample was dissolved in a *n*-hexane-CH_3_CN solution, under N_2_ and magnetic stirring for 5 h before LC-MS/MS analysis.

#### 2.5.2. Method 2

A previously reported method allowing the identification of HtyOle in EVOO from Cassanese cv. was used [14]. Olive pulp (100 g), olive oils (2 g) or dried destoned virgin olive pomace were extracted with methanol/water solution (80/20; *v*/*v*), sonicated for 15 min and then centrifuged for 25 min at 8000 rpm at 5 °C. Supernatants were filtered and diluted for LC-MS/MS analysis.

### 2.6. Preparation of Standard Solutions

The assay of the TyOle was carried out by external standard calibration method. TyOle was dissolved in methanol (10 µg·mL^−1^). This standard solution was diluted with methanol/0.1% formic acid-containing water (70/30; *v*/*v*) and calibration standards were obtained in the concentration range 0.01–0.4 and 0.1–4.0 µg·mL^−1^.

### 2.7. High Performance Liquid Chromatography/Tandem Mass Spectrometry

HPLC analyses were performed by injecting methanol/water extracts (10 µL) in a Discovery C-18 column (particle size: 3 µm; length: 150 mm; i.d.: 4.6 mm) (Merck KGaA, Darmstadt, Germany) using aqueous formic acid (0.1%) solution (A) and methanol (B) as the mobile phase with the following gradient: B increased from 30% to 100% in 10 min, held for 5 min, and then back to 30% in 10 min. Flow rate was 400 µL·min^−1^. TyOle was analyzed in multiple reaction monitoring (MRM) with negative ion mode. The MS parameters, including entrance potential (EP), declustering potential (DP), collision energy (CE) and collision exit potential (CXP) were optimized for the transition chosen.

### 2.8. Method Validation

Two olive oil samples (2 g) were added of different aliquots of TyOle standard solution in centrifuge bottles. Extraction (done in triplicate) was performed as previously described and fortified standard samples (100 and 200 ng·mL^−1^) were obtained. The sample data were analyzed by the external standard method using matrix-matched calibration. The limit of detection (LOD) was calculated as ten times the method noise, including the instrument noise and background signal due to the matrix blank. The limit of quantification (LOQ) was calculated as three times LOD.

### 2.9. Cell Culture

Human immortalized HaCat keratinocytes (CLS Cell Lines Service GmbH, Germany) were cultured in 75 cm^2^ flasks, at 37 °C, 5% CO_2_, in DMEM added of 10% FBS and 1% antibiotics (10,000 μg mL^−1^ streptomycin and 10,000 units∙mL^−1^ penicillin). Cell countings were performed using a Countess Automated Cell Counter (Thermo Fisher Scientifific, Waltham, MA, USA) with Trypan Blue staining. Samples were dissolved in DMSO and diluted in complete medium at the final concentration, with DMSO never exceeding 0.1%. The untreated controls were obtained by adding the same amount of DMSO in the treatments.

### 2.10. Cell Viability Assay

Cells were plated in a 96-well plate at a density of 5.000 cells/well. Cell viability was investigated in response to different concentrations of test compounds (range 2.5–50 µM) after 24 h. An MTT assay was used to estimate cell viability, evaluating the reduction of 3-(4,5-dimethylthiasol-2-yl)-2,5-diphenyltetrazolium bromide (MTT) by mitochondrial succinate dehydrogenase [21]. Absorbance (Abs) was measured by a microtiter plate reader (Synergy H1 by BioTeck, Winooski, VT, USA) at 570 nm (test wavelength, tw) and at 690 nm (reference wavelength, rw). The optical density (OD) was defined as Abs _rw_ − Abs _tw_.

### 2.11. Fluorescent Staining

HaCat cells were grown on glass cover slips inserted into 6-well plates (at a density of 500,000 cells/well). Cells were pre-treated with TyOle (2.5 µM) for 24 h and mitochondrial ROS formation was measured. This was achieved via UV lamp exposure for 30 min at 254 nm (UVC spectrum to avoid vitamin D synthesis). Morphological changes of nuclei and remodeling of cytoskeleton were observed using 4,6-diamidino-2-phenylindole (DAPI) and phalloidin. The potential of the mitochondrial membrane was evaluated by adding 2 µM MitoTracker Red CMXRos dye (*λ* = ex/em = 579/599). Then, cells were fixed with PFA (4%) and the fluorescence was imaged using confocal FV-3000 Olympus microscope and Leica DVM6 microscope (Wetzlar, Germany), as previously described [22].

### 2.12. Superoxide Dismutase Enzymatic Activity

HaCat cells lysate was sonicated and centrifuged at 10,000 rpm for 5 min. The supernatant was used for superoxide dismutase (SOD) enzymatic activity assay. SOD enzymatic activity in cell extracts was assessed as previously reported [23]. Protein concentration in the samples was estimated by Lowry method [24].

The keratinocytes were seeded in 6-well plates (500,000 cells/well) and treated for 6 h with TyOle. After this period, UV lamp exposure at 254 nm (inducer of free radicals) for 30 min was conducted. MitoTracker Red CMXRos (*λ* = ex/em = 579⁄599) was added at a concentration of 2 µM and left to incubate for further 15 min. Fluorescence was read under fluorescence microscopy with the Leica DVM6 microscope.

### 2.13. Statistical Analysis

All experiments were carried out in duplicate in at least three independent experiments. Statistical differences among treatments were assessed by one-way ANOVA followed by Dunnett’s post hoc test. *p* values <0.05 (*), <0.01 (**) and <0.001 (***) were considered as statistically significant.

## 3. Results

### 3.1. HPLC/MS Analyses

#### 3.1.1. Method Validation

**Figure 2:**
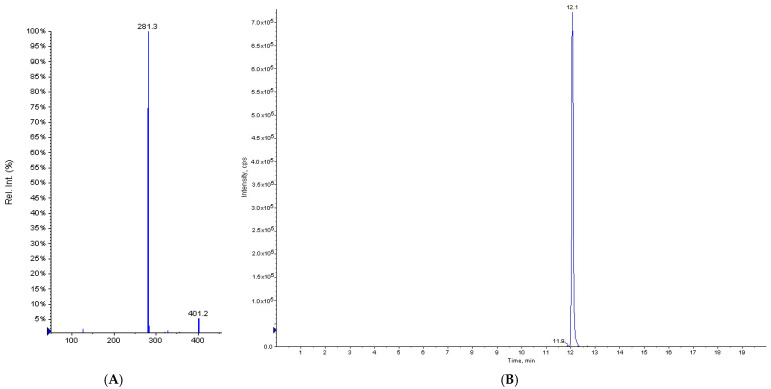
Mass spectrum of TyOle acquired in negative product ion scan (PIS) mode at −25 eV collision energy (**A**). HPLC-MS/MS chromatogram of TyOle. The multiple reaction monitoring (MRM) transition monitored was 401.2 → 281.3 (**B**).

Direct infusion of TyOle into the ion source of the mass spectrometer (flow rate of 10 μL·min^−1^) of a standard solution the analyte was performed to define the optimal conditions for MS parameters. These were optimized to maximize the reading. The mass spectrum of TyOle in product ion scan (PIS) mode showed a pseudomolecular ion at *m*/*z* 401.2 [M − H]^−^ and its main ionic fragment at *m*/*z* 281.3 [oleate anion]^−^ (Figure 2A). The multiple reaction monitoring (MRM) transition chosen and monitored for the assay of TyOle was 401.2 → 281.3. Then, the TyOle standard solution was injected into the HPLC-MS system to define the optimal chromatographic parameters. The best optimized parameters were as follows: source temperature: 400 °C; gas (1): 40 psi; gas (2): 30 psi; dwell time: 400 ms; flow rate: 400 μL·min^−1^. TyOle eluted at 12.1 min (Figure 2B).

Calibration curves were built by least-squares linear regression analysis. The correlation coefficient of linearity was 0.999. Analytical parameters were determined and LOD and LOQ values were found as 0.048 and 0.144 mg·kg^−1^, respectively. Data reported are the mean of three independent analyses.

#### 3.1.2. Identification and Quantification of TyOle in Olive Oils

The occurrence of TyOle was first investigated in olive fruits. However, in our experiments, carried out on the drupes of five different cultivars (Carolea, Cassanese, Coratina, Grossa di Spagna and Nocellara del Belice), TyOle was below the detection limits (Table 1), regardless of the extraction method used. Subsequently, we verified the occurrence of TyOle in olive oils. Indeed, TyOle was identified in all the EVOOs investigated, with values ranging from 0.17 to 1.18 mg·kg^−1^ (Table 1). Interestingly, the concentration of TyOle was found much higher in defective olive oils (DOOs) than EVOOs. Its highest levels were found in lampante oils DOO#2 and DOO#3 that were produced in older campaigns. The extraction method #2 was found to yield higher amount of TyOle with respect to extraction method #1 (7.0 vs. 2.4 mg kg^−1^, respectively), as can be observed in the case of DOO#3. The amount of TyOle was found even higher in dried destoned virgin olive pomace (DDVOP) and in dried destoned virgin olive pomace oil (DDVOPO) (Table 1).

### 3.2. Tyrosyl Oleate Affects Cell Viability of Human Keratinocytes

**Figure 3:**
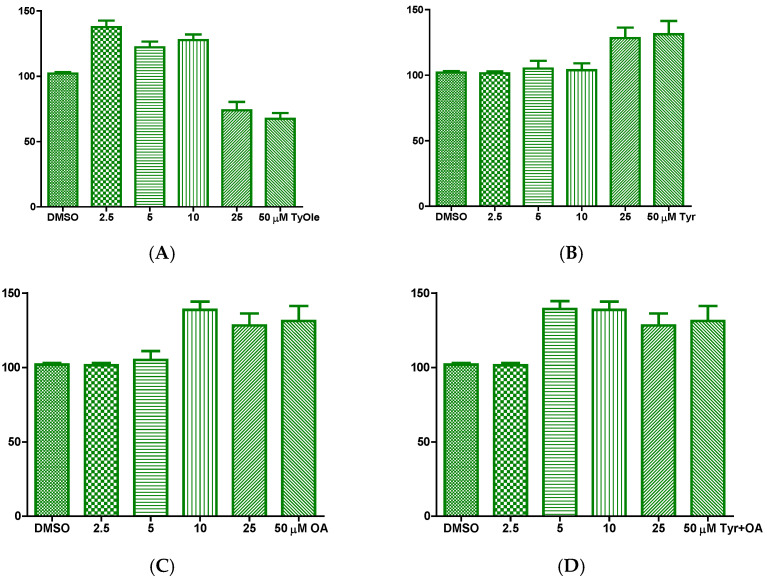
Human keratinocyte cell viability upon 24 h incubation with different concentrations (range 2.5–50 µM) of tyrosyl oleate (TyOle) (**A**), tyrosol (Tyr) (**B**), oleic acid (OA) (**C**), and combination of Tyr and OA (**D**), (* *p* < 0.05; ** *p* < 0.01; *** *p* < 0.001).

Based on our previous results showing that HtyOle possessed antioxidant and tissue regenerating properties in HaCat keratinocytes [14], the potential effects of TyOle were investigated in the same in vitro system. A significant effect on cell vitality was observed when HaCat cells were treated with TyOle. A proliferative effect was recorded when TyOle was added in concentrations up to 10 µM, while cytotoxicity was observed at 25 and 50 µM, as shown in Figure 3A. Tyrosol, oleic acid and their combination were tested as well (Figure 3B–D). We performed time course as well at 12, 24 and 48 h, results did not indicate any difference amongst time-related activity (data not shown). Herein we show 24 h proliferation (Figure 3B–D).

**Figure 4:**
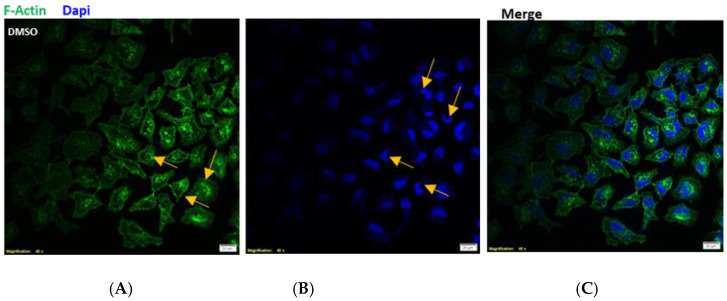

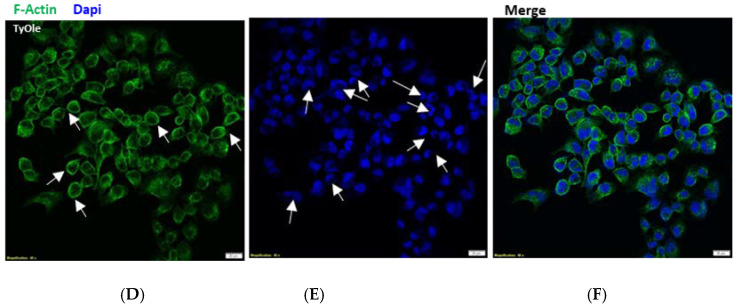
Cytoskeleton remodeling and nuclear lobe formation upon tyrosyl oleate (TyOle) treatment. Representative images of phalloidine (in green) staining in keratinocytes cultured with vehicle (**A**) or with 2.5 µM TyOle (**D**) for 24 h. Nuclei staining with DAPI (in blue) in keratinocytes cultured with vehicle (**B**) or TyOle (**E**). Merged imagine of (**A**–**C**). Merged image of (**D**–**F**). Scale bars: 20 µm.

Based on these results, the lower effective concentration of TyOle (2.5 µM) was selected for further experiments. As it is shown in Figure 4, cytoskeletal remodeling was observed in keratinocytes cultured with TyOle (panel D). Figure 4A indicates that the filaments of F actin showed a centromedian cellular localization highlighted by yellow arrows. Upon treatment with TyOle for 24 h, the isoform F of the actin stained with phalloidine showed a cortical localization close to the plasma membrane as shown in panel D highlighted by white arrows. In Figure 4, an increased nuclear lobe formation in keratinocytes cultured with TyOle (panel E) compared to the vehicle alone (panel B) is shown and highlighted by white arrows. Merged images of both conditions are shown in Figure 4C,F, respectively. Moreover, we also investigated the possibility that TyOle could modulate the antioxidant machinery of human keratinocytes.

**Figure 5:**
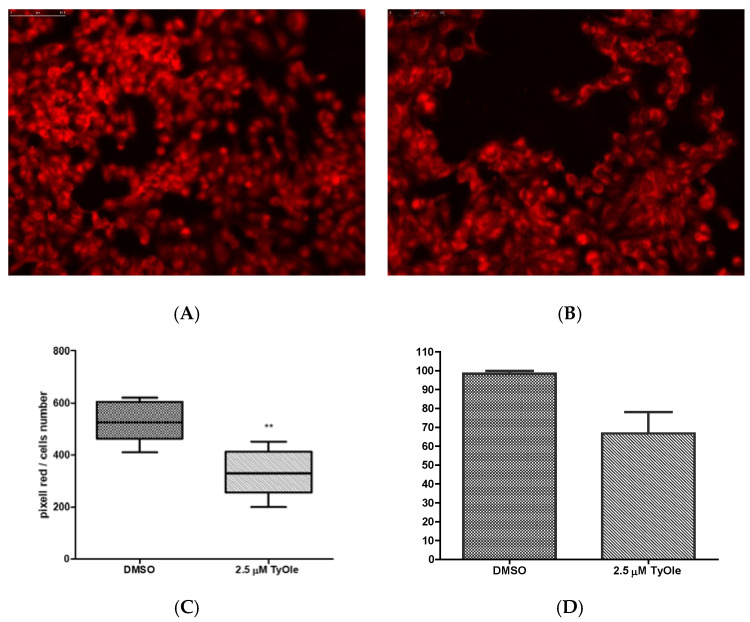
Mitochondrial ROS staining in human keratinocytes treated with vehicle (DMSO) (**A**) or 2.5 µM of tyrosyl oleate (TyOle) (**B**). Pixels for red stained keratinocytes (**C**). Enzymatic activity of superoxide dismutase (SOD) (**D**). Data are reported as the means ± S.D. of three independent experiments each done in duplicate (* *p* < 0.01, ** *p* < 0.05 versus control).

To evaluate TyOle potential effect on mitochondrial ROS formation, MitoTracker Red CMXRos staining was carried out. Keratinocytes cultured for 24 h with TyOle and then exposed to UV (Figure 5B) were characterized by a reduction of stain intensity with respect to the control (treated with vehicle and then exposure to UV, Figure 5A). Figure 5C shows the average values of cell area in pixels for red stained keratinocytes. Furthermore, in our set of experiments, superoxide dismutase (SOD) activity was decreased by TyOle (Figure 5D).

## 4. Discussion

In the last decades, a growing interest has been observed for lipophilic derivatives of phenolics, particularly for those bearing a lipid moiety (lipophenols) [25,26,27]. Phenolic esters bearing a fatty acid acyl chain have been found to retain and even increase the antioxidant properties of the parent compound [28,29,30,31,32,33,34,35,36,37]. Moreover, they showed higher metabolic stability and bioavailability due to their higher lipophilicity with respect to corresponding phenolics that undergo fast elimination in humans, mainly due to their hydrophilic character [38,39,40,41]. Moreover, some phenolic fatty esters have been synthesized in order to obtain a potential emulsifier and stabilizer of oil matrices [42,43,44,45,46,47,48,49,50].

Despite these compounds having been mainly known as semi-synthetic phenolic derivatives characterized by enhanced antioxidant activities, hydroxytyrosyl oleate (HtyOle, Figure 2), a derivative of Hty containing an oleic acid residue, has been recently found in olive oil [13]. Moreover, HtyOle was identified in a monovarietal EVOO (cultivar: Carolea) produced in the campaign 2017/2018 and its concentration was found as 4.9 mg kg^−1^ of oil [14]. HtyOle has also been found in olive oil byproducts, namely pomace and olive mill waste waters (OMWWs) [15]. The congener tyrosyl oleate (TyOle, Figure 2) has been first isolated and identified in the fruits of Cassanese cultivar [17]. This finding was not confirmed in the present study, as we were not able to detect TyOle in the olive fruits. By contrary, TyOle was found in olive oils as well as in the by-products of olive oil industry, such as dried destoned virgin olive pomace (DDVOP) and the corresponding oil. This molecule is likely formed following an esterification reaction catalyzed by an appropriate enzyme activated by the pressing process, starting from the precursors, oleic acid and tyrosol. Furthermore, TyOle was found in higher levels in low-quality and defective oils. This could suggest that the presence of this molecule is correlated to the increase in the defect and to the increase in the concentration of precursors both during the rancidity, crushing and kneading processes. Further experiments with a larger body of samples characterized by differences in cultivar, origin, processing, and age will be necessary to verify the possibility that TyOle could represent a marker for the quality of oils.

Antioxidants are crucial to reduce the effects of oxidative stress on the skin and therefore for the prevention or reduction of skin aging. The use of naturally occurring antioxidants is growing as there is an increase in the demand of consumers for natural and more environmentally friendly products that are considered safer and healthier replacer of synthetic compounds [51]. In this context, lipophilic derivatives of phenolic compounds represent a valuable class for their increased capacity of skin penetration due to their lipophilicity character that make them more advantageous with respect to the corresponding hydrophilic parent compounds, thus increasing their antimicrobial, antioxidant, or anti-ageing activities [16,17,18,52]. In our work, TyOle was found to induce an increase in cell viability (up to 10 µM) and the cytoskeletal remodeling and keratinocytes terminal differentiation, which is characterized by nuclear lobe formation. Those are hallmarks of skin regeneration [19]. Furthermore, the presence of TyOle led to a reduction in mitochondrial ROS production supported by a diminishing of SOD activity. ROS balance is crucial in all tissues, including skin. On the other hand, ROS unbalance due to higher levels of oxidative stress and reduced antioxidant defenses has been correlated to skin–dermal dysfunction [53,54]. In this view, ROS clearance is promoted by antioxidant enzymes [55]. Among these enzymes, SOD promotes the dismutation of superoxide anion with formation of oxygen and hydrogen peroxide within the mitochondria. Therefore, the redox state of this compartment is strictly linked to SOD activity [56]. Our experiments showed that SOD activity is decreased upon TyOle treatment. This indicates that TyOle plays as a free radical scavenger, yielding a low cellular redox state. Under conditions of oxidative stress, the cells use antioxidant enzymes to protect themselves [57]. On the other hand, the enzymatic activity is reduced when free radicals and lipid peroxidation are decreased [58]. Indeed, when cellular redox state is low, detoxifications mediated by antioxidant enzymes are found low as well [59]. In the complex picture of human skin regeneration, our results are of importance also from a nutritional perspective since for skin healing care research should focus on the biochemical pathways involved in, and on the relationships with a specific supplement use [60].

## 5. Conclusions

In conclusion, in this work tyrosyl oleate (TyOle) was found as a novel lipophenol in olive oil and in by-products. TyOle was identified and quantified in all the oil samples investigated while it was under the detection limit in olive fruits. Interestingly, TyOle was found in higher concentration in defective or low-quality oils than in EVOOs. The reaction of tyrosol and oleic acid leading to the formation of TyOle is likely occurring during crushing and kneading processes when suitable enzymes are activated. TyOle concentration increases with rancidity due to storage for a long period in not appropriate condition when the concentration of the free precursors increase. Further research will be devoted to investigating the potential of this compound as a marker of quality in olive oils. Moreover, we showed that TyOle induced an increase in the viability of HaCat cells and cytoskeletal remodeling and could represent a valuable adjuvant against skin oxidation.

## Figures and Tables

**Table 1:** Amount of tyrosyl oleate (TyOle) in extracts from olives and olive oils ^1^.

Sample	Cultivar	Campaign	ExtractionMethod	TyOle(mg·kg^−1^)
Fruit#1	Carolea	2017/2018	1 and 2	<LOD
Fruit#2	Cassanese	2017/2018	1 and 2	<LOD
Fruit#3	Coratina	2017/2018	1 and 2	<LOD
Fruit#4	Grossa di Spagna	2017/2018	1 and 2	<LOD
Fruit#5	Nocellara del Belice	2017/2018	1 and 2	<LOD
EVOO#1	Carolea	2017/2018	2	1.18 ± 0.08
EVOO#2	Dolce di Rossano	2017/2018	2	0.7 ± 0.2
EVOO#3	Nocellara del Belice	2017/2018	2	0.17 ± 0.08
DOO#1	Blend	2016/2017	2	1.0 ± 0.3
DOO#2	Blend	2014/2015	2	5.0 ± 0.8
DOO#3	Blend	2014/2015	2	7.0 ± 0.4
DOO#3	Blend	2014/2015	1	2.4 ± 0.1
DDVOP		2017/2018	2	8.2 ± 0.2
DDVOPO		2017/2018	2	33.2 ± 0.5

^1^ EVOO: Extra virgin olive oil; DOO: Defective olive oil; DDVOP: Dried destoned virgin olive pomace; DDVOPO: Dried destoned virgin olive pomace oil. Data are reported as mean ± S.D. (*n* = 3).

## Data Availability

Data is contained within the article.
